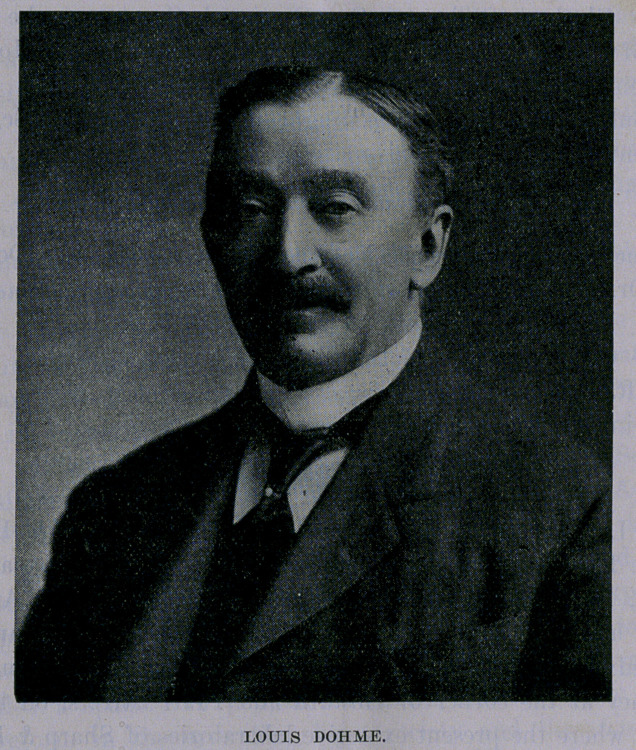# Mr. Louis Dohme

**Published:** 1911-01

**Authors:** 


Mr. Louis Dohme, one of the founders of Sharp & Dohme, died at Baltimore on December 12, 1910. Mr. Dohme was born July 6, 1837, at Obernkirchen, Germany. He came to America at the age of 15, landing at Baltimore, where he became acquainted with Mr. A. P. Sharp, who at that time conducted a pharmacy at the corner of Howard and Pratt Streets, on the exact site where the present extensive laboratories of Sharp & Dohme are located. Mr. Dohme even at that early age had a pronounced liking for the drug business, and he therefore eagerly accepted the position of assistant, offered to him by Mr. Sharp and thus began the associations which eventually developed into the foundation of one of the most widely known firms of manufacturing pharmacists in this country. Mr. Dohme studied pharmacy in all of its practical details under the skilled tutelage of Mr. Sharp, and after attending the prescribed course of lectures at the Maryland College of Pharmacy was graduated in 1857. In later years he became the President of his alma mater and as such is still remembered by a host of alumni of that well known institution.

Mr. Dohme served on several U. S. P. Revision Committees, and his work was always painstaking and accurate, hence valuable.

The firm of Sharp & Dohme was established in 1860. After Mr. Charles E. Dohme became connected with the business, Sharp & Dohme began the manufacture of a line of pharmaceuticals characterized then as now by reliability.

For several years Mr. Louis Dohme traveled through the West and South at intervals, and by introducing the firm's products to the wholesale and retail drug trade laid the foundation for the

LOUIS DOHME.

large patronage now enjoyed by Sharp & Dohme in that section.

The firm was incorporated in 1892, Mr. Louis Dohme becoming President; Mr. Chas. E. Dohme, Vice-President, and Mr. Ernest Stauffen, Secretary and Treasurer. The business was reorganized and the capital stock increased in 1903, the same officers being elected, together with Dr. A. R. L. Dohme, the only son of Charles E., as Second Vice-President.

Dr. A. R. L. Dohme for many years has been in general charge of the laboratories. Mr. Stauffen, who joined the firm in 1876, has since then been in charge of the general business department, and since 1893 has managed the firm's business from their general offices at 41 John Street, New York.

Mr. Louis Dohme has not taken any active interest in business during recent years, preferring to place all responsibility upon the shoulders of the younger, more active members of the corporation, and to spend a part of each year abroad.

Mr. Dohme had a charming personality and his affability readily won for him a large circle of friends. He was never married, and for many years had his home with his brother, Charles E., who with his family, his brother William and a number of nephews and nieces survive him.

Mr. Dohme's death, owing to his keen foresight in years ago transferring the active management of his business to his trusted associates, will not affect the business of Sharp & Dohme, a business with which he was closely associated during the major portion of his life. He was buried at Baltimore on December 15th.

## Figures and Tables

**Figure f1:**